# Epidemiology and Outcomes of Takotsubo Syndrome in Hospitalizations With Systemic Sclerosis

**DOI:** 10.7759/cureus.9925

**Published:** 2020-08-21

**Authors:** Zainab Gandhi, Hee Kong Fong, Pritika Manaktala, Faizan A Malik, Sejal Savani, Neelesh Gupta, Rupak Desai

**Affiliations:** 1 Internal Medicine, Geisinger Community Medical Center, Scranton, USA; 2 Cardiovascular Medicine, University of California Davis Medical Center, Sacramento, USA; 3 Internal Medicine, Canton Medical Education Foundation, Canton, USA; 4 Internal Medicine, Texas Tech University Health Sciences Center at Permian Basin, Odessa, USA; 5 Public Health, New York University, New York, USA; 6 Internal Medicine, Nazareth Hospital, Trinity Health Mid-Atlantic, Philadelphia, USA; 7 Cardiology, Atlanta Veterans Affairs Medical Center, Decatur, USA

**Keywords:** takotsubo syndrome, systemic sclerosis, outcomes, epidemiology, takotsubo cardiomyopathy, scleroderma, stress induced cardiomyopathy, apical ballooning syndrome, broken heart syndrome

## Abstract

Background

Systemic Sclerosis (SSc) is associated with chronic inflammation which leads to macrophage activation and thus vascular insult and fibrosis. Macrophage activation is shown to precede Takotsubo syndrome (TTS) which may be a common pathophysiologic link to SSc.

Methods

We queried the National Inpatient Sample (2008-2014) for adult SSc-related hospitalizations and TTS using relevant International Classification of Diseases Clinical Modification, 9th Revision codes. We assessed the prevalence and trends in TTS during this time. We further assessed demographics, comorbidities, and outcomes were in SSc with and without TTS. The primary outcomes of the analysis were all-cause mortality and in-hospital complications including cardiac arrest and acute myocardial infarction (AMI), arrhythmias, and venous thromboembolism, and stroke.

Results

A total of 213,728 SSc-related hospitalizations were found, of which 357 experienced TTS (0.2%) with rising trends in TTS from 2008-2014 (0.06% to 0.3%, relative increase of 24%, p_trend_<0.001). The TTS cohort was older (median age 68 vs 62 years), with 92.8% females and 80.1% white adults with TTS (p<0.001). Co-morbidities were higher in the TTS cohort including hypertension (62.2% vs. 51.5%, p<0.001), dyslipidemia (41.5% vs. 22.8, p<0.001), smoking (28.9% vs. 20.1%, p<0.001), peripheral vascular disease (17.8% vs. 9.1%, p<0.001), uncomplicated diabetes (18.1% vs. 11.9%, p<0.001). The all-cause in-hospital mortality (11% vs. 4.6%; adjusted odds ratio=1.82, 95% confidence interval: 1.21-2.72, p<0.005), cardiovascular complications like AMI (29% vs. 2.9%,p<0.001), arrhythmias (38.9% vs. 21.5%, p<0.001), and median length of stay [6 vs. 4 days] were significantly higher in the TTS cohort as compared to the non-TTS cohort.

Conclusion

This analysis revealed a nearly 10 times higher prevalence of TTS in SSc-related hospitalizations compared to the general inpatient population. Concomitant TTS occurrence in SSc-related hospitalizations led to nearly two times higher odds of all-cause mortality. Cardiovascular co-morbidities in SSc may increase the risk of TTS and worsened outcomes.

## Introduction

Systemic sclerosis (SSc) is a chronic autoimmune disease that is characterized by vascular endothelial dysfunction and fibrosis of the internal organs and skin. It is mainly associated with Raynaud phenomenon, pulmonary arterial hypertension, and scleroderma renal crisis [1]. The pathogenesis of SSc involves chronic inflammation, followed by vascular insult and thus fibrosis. Macrophage activation secondary to chronic inflammation can lead to the production of mixed pro-inflammatory and anti-inflammatory cytokines and chemokines such as interleukin-4, interleukin-3, tumor necrosis factor, interleukin-6, transforming growth factor-beta, and chemokine (C-C motif) ligand 2 (CCL2) which mediate the fibrosis in SSc [2]. Takotsubo syndrome (TTS) has shown to be preceded by prolonged macrophage inflammatory infiltration and pro-inflammatory cytokines [3]. Melchiorre et al. in 2008 reported the first case of TTS in SSc patients suspecting microvascular injury, myocardial fibrosis, and Raynaud phenomenon, which contributes to the development of TTS [4].

## Materials and methods

In this first large-scale analysis, we sought to understand the epidemiology and outcomes of TTS in patients admitted with SSc in the United States. We conducted a retrospective analysis on National Inpatient Sample (2008-2014) with the International Classification of Diseases Clinical Modification, 9th Revision codes of TTS (429.83) and SSc (710.1) to study the SSc-related admissions with vs without TTS.

The study population involved inpatient hospitalizations in the National Inpatient Sample from 2008 to 2014. We assessed the in-hospital outcomes of SSc-related admissions with TTS and without TTS using multiple study variables. Demographics like age, sex, and race were considered. Co-morbidities and in-hospital outcomes associated with SSc-related admissions with TTS and without TTS were assessed.

The primary outcomes for the analysis were all-cause mortality and in-hospital complications including cardiac arrest and acute myocardial infarction (AMI), arrhythmias, and venous thromboembolism, and stroke.

Statistical Package for the Social Sciences (SPSS), version 24 (IBM Corp, Armonk, NY) was utilized to complete statistical analyses using defined strata/cluster designs. It was compared using the Chi-square test and Mann Whitney U test for categorical and continuous variables, respectively.

## Results

The prevalence of TTS in SSc-related admissions was 0.2% (n=357) amongst the total SSc-related admissions (n= 213,728) from 2008 to 2014. The SSc-related admissions with TTS often showed elderly [68 (60-75) vs 62 (52-72) yrs; p<0.001], white (80.1% vs 69.4%; p<0.001), female (90.8% v/s 80.1% p<0.001) patients admitted for non-elective procedures (87.2 % vs 81.8%; p=0.01) as compared to the non-TTS cohort.

The prevalence of comorbidities including peripheral vascular disease (17.8% vs 9.1%; p<0.001), hypertension (62.2% vs 51.5%; p<0.001), smoking (28.95 vs 20.1%; p<0.001) and coagulopathy (11.4% vs 6.1%; p<0.001) was higher in SSC-related admissions with TTS cohort vs without TTS. Higher prevalence of chronic pulmonary disease (28.8% vs 22.2%; p<0.001) and pulmonary circulation disorders (21.1% vs 11.0%; p=0.134) was observed in SSc-related admissions with TTS vs without TTS.

Overall all-cause mortality in the SSc-TTS cohort was more than two times higher (11% vs 4.6%, p<0.001) as compared non-TTS cohort. The SSc-TTS cohort had higher burden of in-hospital arrhythmias (38.9% vs 21.5%; p<0.001), acute myocardial infarction (29% vs 2.9%; p<0.001), venous thromboembolism (6.9% vs 3.1%; p<0.001), stroke (5.5% vs 1.7%; p<0.001) and in-hospital cardiac arrest (4.3% vs 1.7%; p<0.001) as compared to non-TTS cohort (Table 1).

**Table 1:** Systemic sclerosis-related admissions with versus without Takotsubo syndrome P<0.05 indicates statistical significance. IQR, interquartile range; MI, myocardial infarction; PCI, percutaneous coronary intervention; CABG, coronary artery bypass grafting; SNF, skilled nursing facility; ICF, intermediate care facility. Note: Cell sizes less than 11 were indicated by NA as per the privacy guidelines under the Healthcare Cost and Utilization Project (https://www.hcup-us.ahrq.gov/db/publishing.jsp).

Variable		No Takotsubo Syndrome (n=213,372)		Takotsubo Syndrome (n=357)		P
		Number	%	Number	%	
Age at admission	Median [IQR]	62 [52–72]		68 [60–75]		<0.001
	18-44	27,441	12.9%	NA	NA	<0.001
	45-64	90,129	42.2%	138	38.7%	
	>=65	95,801	44.9%	219	61.3%	
Sex	Male	33,863	15.9%	26	7.2%	<0.001
	Female	179,493	84.1%	331	92.8%	
Race	White	133,668	69.4%	262	80.1%	<0.001
	African American	30,173	15.7%	20	6.1%	
	Hispanic	18,948	9.8%	15	4.6%	
	Asian or Pacific Islander	3,419	1.8%	15	4.7%	
	Native American	1,573	0.8%	NA	NA	
	Others	4,926	2.6%	15	4.6%	
Type of admission	Non-elective	174,215	81.8%	311	87.2%	0.01
	Elective	38,664	18.2%	46	12.8%	
Comorbidities						
	Alcohol abuse	3,812	1.8%	NA	NA	0.582
	Coagulopathy	13,021	6.1%	41	11.4%	<0.001
	Deficiency anemias	60,887	28.5%	123	34.4%	0.013
	Chronic pulmonary disease	47,345	22.2%	103	28.8%	0.002
	Depression	31,345	14.7%	59	16.5%	0.328
	Diabetes, uncomplicated	25,327	11.9%	65	18.1%	<0.001
	Diabetes with chronic complications	6,078	2.8%	NA	NA	0.001
	Drug abuse	4,875	2.3%	NA	NA	0.514
	Hypertension	109,961	51.5%	222	62.2%	<0.001
	Liver disease	9,672	4.5%	20	5.7%	0.332
	Obesity	12,831	6.0%	NA	NA	0.011
	Peripheral vascular disorders	19,496	9.1%	63	17.8%	<0.001
	Pulmonary circulation disorders	38,324	18.0%	75	21.1%	0.134
	Renal failure	33,362	15.6%	51	14.2%	0.483
	Solid tumor without metastasis	3,359	1.6%	20	5.7%	<0.001
	Valvular heart disease	14,599	6.8%	34	9.6%	0.045
	Dyslipidemia	48,727	22.8%	148	41.5%	<0.001
	Previous MI/PCI/CABG	20,025	9.4%	63	17.8%	<0.001
	Smoking	42,914	20.1%	103	28.9%	<0.001
Outcomes						
	All-cause mortality	9,795	4.6%	39	11.0%	<0.001
	Arrhythmia	45,891	21.5%	139	38.9%	<0.001
	Acute myocardial infarction	6,229	2.9%	104	29.0%	<0.001
	Venous thromboembolic events	6,689	3.1%	25	6.9%	<0.001
	Stroke	3,685	1.7%	20	5.5%	<0.001
	In-hospital cardiac arrest	3,592	1.7%	15	4.3%	<0.001
Disposition						<0.001
	Routine	121,770	57.1%	157	44.0%	
	Transfer to short-term hospitals	6,096	2.9%	15	4.2%	
	Transfer to other (SNF, ICF)	34,140	16.0%	95	26.7%	
Length of stay, median [IQR], days		4 [2–7]		6 [3–12]		<0.001
Total charges, median [IQR], $		27,952 [15085–53704]		62,560 [28956–115320]		<0.001

## Discussion

In our analysis, we found that SSc-related admissions with TTS were found to be 0.2%, which is nearly 10 times higher than the prevalence of TTS (0.02%) reported in the general inpatient population in the United States [5]. SSc-related admissions with TTS were seen more commonly in the elderly, white, and females, and patients with pre-existing comorbidities like hypertension, peripheral vascular disease, smoking, and coagulopathy. The observation of prevalence in the older female patients can be secondary to the post-menopausal state with decreased estrogen levels, as estrogen has shown benefit in coronary blood flow and preventing endothelial dysfunction [1]. The female predominance associated with both of these conditions could be the reason [1].

Endothelial dysfunction possibly contributed by smoking to the SSc-related TTS cohort. In addition, the pathophysiological process of SSc with increased endothelin and decreased nitric oxide and prostacyclin levels lead to increased pro-fibrotic factors and vascular fibrosis in the SSc-related TTS cohort. The compromise of pulmonary vascular endothelium in SSc, eventually leads to an increased prevalence of the comorbidity. There was a higher prevalence of in-hospital complications of venous thromboembolism and stroke in the TTS-SSc cohort. Increased coagulopathy due to platelet activation and enhanced coagulation disorders are also associated with SSC-related admissions with the TTS cohort [1,6].

Furthermore, in SSc-related admissions with TTS, we found the all-cause mortality was double in comparison to the non-TTS cohort. Secondary outcomes like in-hospital arrhythmias, acute myocardial infarction, in-hospital cardiac arrest also showed a higher burden in the TTS cohort in comparison to the non-TTS cohort. Accelerated atherosclerosis due to increased immune response, and systemic inflammation leading to premature atherosclerosis is a widely known mechanism for SSc. Vasospasm in TTS contributes to exacerbating ischemia and myocardial fibrosis, which could be a possible explanation for the observed higher frequency of AMI in the TTS cohort. Arrhythmias are secondary to the conduction system and myocardial fibrosis, secondary to endothelial dysfunction in SSc [7]. Due to the higher burden of worse outcomes in the TTS cohort, it can lead to higher utilization of health care resources with increased length of stay as seen in our analysis. It can lead to higher utilization of financial, material and human resources in the healthcare system.

Limitations

Potential limitations of this study include billing and coding issues due to administrative data collection, lack of longitudinal follow-up, lack of data on the primary cause of death, lack of medication history and non-availability of laboratory results. Furthermore, there could be a possibility of residual confounders in the univariate analysis as the small sample of SSc hospitalizations did not allow us to explore comprehensive multivariable analysis. Despite these limitations, nationally representative cohorts of TTS and SSc enabled us to perform this analysis on one of the understudied subjects.

## Conclusions

In conclusion, the prevalence of TTS in SSc-related admissions was 0.2%. SSc-related admissions showed higher than two times the all-cause mortality, 10 times higher frequency of acute myocardial infarction and higher frequency of arrhythmias, venous thromboembolism, stroke, and acute cardiac arrest with statistical significance. This study highlights the possible relation of worse prognosis for SSc-related admissions with an episode of TTS versus without TTS.
